# Mechanism of Cell Wall Polysaccharides Modification in Harvested ‘Shatangju’ Mandarin (*Citrus reticulate* Blanco) Fruit Caused by *Penicillium italicum*

**DOI:** 10.3390/biom9040160

**Published:** 2019-04-24

**Authors:** Taotao Li, Dingding Shi, Qixian Wu, Chunxiao Yin, Fengjun Li, Youxia Shan, Xuewu Duan, Yueming Jiang

**Affiliations:** 1 Guangdong Provincial Key Laboratory of Applied Botany, South China Botanical Garden, Chinese Academy of Sciences, Guangzhou 510650, China; Sdd6531@scbg.ac.cn (D.S.); wuqixian@scbg.ac.cn (Q.W.); lifengjun@scbg.ac.cn (F.L.); shanyouxia@126.com (Y.S.); xwduan@scbg.ac.cn (X.D.); 2 Key Laboratory of Post-Harvest Handling of Fruits, Ministry of Agriculture, Guangzhou 510650, China; 3 University of Chinese Academy of Sciences, Beijing 100039, China; 4 Long Ping Branch, Graduate School of Hunan University, Changsha 410125, China; chunxiaoyin@scbg.ac.cn

**Keywords:** citrus fruit, reactive oxygen species (ROS), gene, polysaccharides, *Penicillium*

## Abstract

Modification of cell wall polysaccharide in the plant plays an important role in response to fungi infection. However, the mechanism of fungi infection on cell wall modification need further clarification. In this study, the effects of *Penicillium italicum* inoculation on ‘shatangju’ mandarin disease development and the potential mechanism of cell wall polysaccharides modification caused by *P. italicum* were investigated. Compared to the control fruit, *P. italicum* infection modified the cell wall polysaccharides, indicated by water-soluble pectin (WSP), acid-soluble pectin (ASP), hemicellulose and lignin contents change. *P. italicum* infection enhanced the activities of polygalacturonase (PG), pectin methylesterase (PME), and the expression levels of *xyloglucanendotransglucosylase/hydrolase (XTH)* and *expansin*, which might contribute to cell wall disassembly and cellular integrity damage. Additionally, higher accumulation of reactive oxygen species (ROS) via decreasing antioxidant metabolites and the activities of antioxidant enzymes including superoxide dismutase (SOD), catalase (CAT), and ascorbate peroxidase (APX) also contributed to the cell wall polysaccharides modification. Meanwhile, the gene expression levels of hydroxyproline-rich glycoprotein (HRGP) and germin-like protein (GLP) were inhibited by pathogen infection. Altogether, these findings suggested that cell wall degradation/modification caused by non-enzymatic and enzymatic factors was an important strategy for *P. italicum* to infect ‘shatangju’ mandarin.

## 1. Introduction

Citrus is an important fruit and widely grown worldwide. Citrus is one of the major sources of bioactive compounds including vitamins, carotenoids, fiber, flavonoids and other phenolics, as well as essential minerals [1]. Approximately 25% of citrus fruits are lost due to postharvest decay caused by fungal infections [2,3]. *Penicillium digitatum* and *Penicillium italicum* are the most important pathogens responsible for significant postharvest losses in citrus fruit [4,5]. *P. italicum*, as one of the most important fungal pathogens, triggers blue mold diseases, and causes decay of citrus fruit worldwide [2,6]. Though the optimum temperature for germination and growth of *P. italicum* is 25 °C, it can also be active at 4–30 °C or even at 0 °C; but no growth at 37 °C [5]. In addition, *P. italicum* can germinate and grow at water activities (aw) of 0.87 [5]. Currently, fungicides are widely used to control postharvest fungal disease. However, the use of fungicides caused public concern over human health and environmental risks [7]. Additionally, the development of resistant fungi strains caused by extensive use of chemical fungicides results in limited efficacy [4]. Papoutsis et al. [4] reviewed some non-chemical postharvest treatments (irradiations, biocontrol agents, natural compounds, hot water treatment, and salts), which might provide an alternative to synthetic fungicides to prevent postharvest decay caused by *P. italicum*. Importantly, Palou et al. [8] suggested that the more we learn about the host-pathogen interactions, more effective methods to control fruit disease will emerge. Therefore, an improved understanding of infection mechanism of pathogen may help to develop alternative strategies to reduce postharvest losses of fruit.

Cell wall-associated plant defense is vital in basal resistance [9]. The cell wall is believed to be a critical factor in plant response to fungi infection by providing a physical barrier between pathogens and the internal contents of the plant cells [10]. The solubilization and depolymerization of the cell wall constituents would facilitate postharvest pathogens infections to increase postharvest decay [11]. Shi et al. [11] also reported that the use of graft copolymer could suppress rotting of grapefruit by inhibiting solubilization of cell wall polysaccharides. In previous research, we found that the degradation of cell wall polysaccharides occurred during ‘shatangju’ mandarin fruit senescence, which might be related to decreased disease resistance [12]. Whereas, the mechanism of fungi infection on cell wall modulation still need to be further clarified.

Cell wall-related enzymes, such as polygalacturonase (PG), pectinmethylesterase (PME), and xyloglucan endotransglucosylase/hydrolase (XTH), play important roles in polysaccharide modification and enzymatic disassembly of the plant cell wall [13,14]. Meanwhile, non-enzymatic modification of polysaccharide also leads to the degradation of cell wall polysaccharides [15,16,17,18,19]. Besides, cell wall-related proteins such as hydroxyproline-rich glycoprotein (HRGPs) and expansin were also reported to be involved in the cell wall architecture [20,21]. In all, cell wall degradation caused by enzymatic and non-enzymatic factors were well reported to be involved in fruit ripening [16,22,23]. However, rare effort to date has been put into fungi induced cell wall change and its relation to disease development of harvested citrus fruit.

Citrus fruit can be classified into tight-skin citrus and loose-skin citrus [24]. ‘Shatangju’ mandarin (*Citrus reticulate* Blanco) is a typical loose-skin and a Chinese specialty citrus [25]. Due to its outstanding features including thin-skin, easy-peeling, good-looking and sweet fruit flesh, ‘shatangju’ mandarin has become the major variety which could bring more profit to local farmers [26]. Also, ‘shatangju’ mandarin is vulnerable to fungal infection, such as *P. italicum*.

To acquire more information on the infection mechanism of *P. italicum* on citrus fruit, we focused on the effects of pathogen infection on cell wall modification through enzymatic and non-enzymatic aspects. This study aims to clarify the mechanism of *P. italicum* infection inducing disease development of stored ‘shatangju’ mandarin fruit in a perspective of cell wall metabolism. Our results can provide a theoretical basis for the development of alternative fungicides to control citrus diseases.

## 2. Materials and Methods 

### 2.1. Fruit Sample and Treatments

Mandarin (*Citrus reticulata* Blanco ‘Shatangju’) fruit were harvested at approximately 240 d after blooming from a commercial orchard in Conghua, Guangdong Province, China. Fruit were selected for uniformity of shape, color, and size, with a total soluble solid of approximately 11.0% as described by our previous research [12]. The selected fruit were soaked in 0.1% Sportak fungicide solution (Prochloraz) for 3 min, then washed with water. After being air-dried for 2 h, fruit were used for pathogen inoculation.

*P. italicum* strain was isolated from naturally infected sweet ‘shatangju’ mandarin fruits and stored in −80 °C with glycerol. The isolate was cultured on potato dextrose agar (PDA) for 6 days at 28 °C before use. Then, the spores were washed with sterile distilled water and the conidial suspension was adjusted to 1 × 10^6^ cells/ml (conidia per milliliter). The inoculations were performed according to Deng et al. [27] with 450 fruit for each inoculation. The fruit inoculated with *P. italicum* were designated as Pi treatment while fruit pipetted with sterile distilled water were set as the control group. After inoculation, the fruit were packaged in plastic bags (thirty fruit per bag) and transferred to storage at 25 °C, 90–95% relative humidity (RH). Pericarp around the inoculation site (1 cm around the inoculation site) were taken at day 0, day 1, day 3, and day 5. The samples were immediately frozen with liquid nitrogen and stored in −80 °C refrigerator for further use. Three independent biological replicates with 150 fruit for per replicate were conducted with thirty fruit per replicate.

### 2.2. Cell Wall Component Analysis

Three grams of tissue from thirty mandarin fruit peel were used for pectin extraction according to Cheng et al. [28] with minor modification. Then water-soluble pectin (WSP) and acid-soluble pectin (ASP) contents were measured using the method described by Zhao et al. [29]. 

For hemicellulose content analysis, 3 g of peel tissue from thirty mandarin fruit was used for hemicellulose extraction according to our previous method [28]. Then, hemicellulose content was measured according to the method described by Wang et al. [30].

Lignin content was measured using 3 g of tissue from thirty mandarin fruit peel by the method of Deng et al. [31]. 

### 2.3. ROS and Ascorbic Acid (AsA) Content Measurement

ROS contents measurements were conducted using our previous research [32] with 1 g of peel tissue from thirty mandarin fruit. Ascorbic acid (AsA) content was measured according to Sun et al. [33] using 1 g of peel tissue from thirty mandarin fruit.

### 2.4. Enzyme Activities Assays

Superoxide dismutase (SOD, EC 1.15.1.1) and catalase (CAT, EC 1.11.1.6) activities were analyzed with 3 g of peel powders, ground with liquid nitrogen using FW-100 grinder, from thirty mandarin fruit using the method described in our previous research [32]. 

Ascorbate peroxidase (APX, EC 1.11.1.11) activity was measured according to the APX Enzyme Activity Assay Kit (Jiancheng Bioengineering, Nanjing, China) using 3 g of peel powders from thirty mandarin fruit. The absorbance at 290 nm was recorded to calculate the APX activity. One unit (U) of APX activity was defined as enzyme required to oxidize 1μmol AsA per min. 

Three gams of mandarin peel samples from thirty fruit were used for polygalacturonase (PG, EC 3.2.1.15) and pectin methylesterase (PME, EC 3.1.1.11) activities measurement using the approaches described by Zhao et al. [34].

### 2.5. RNA Isolation and Real-Time Quantitative PCR

Total RNA of each sample was isolated using 5 g of peel powders from thirty fruit according to the method described by our previous research [32]. After cDNA synthesis, PrimeScriptTM RT Master Mix (TaKaRa-RR036A, Dalian, China) was used for gene expression level analysis according to our previous method [32]. The primers for selected genes, which were shown in Table S1, were designed using Primer Premier 6.0 software (Premier, Canada). *CiActin* was used as the endogenous control to normalize the content of cDNA.

### 2.6. Statistical Analysis

The data were expressed as the mean values of three biological replicates. SPSS version 7.5 was used to test above data by one-way analysis of variance (ANOVA) with the least significant differences at 0.05 level.

## 3. Results

### 3.1. Effects of *P. italicum* Infection on Pectin and Lignin Contents of Mandarin Peel

**Figure 1:**
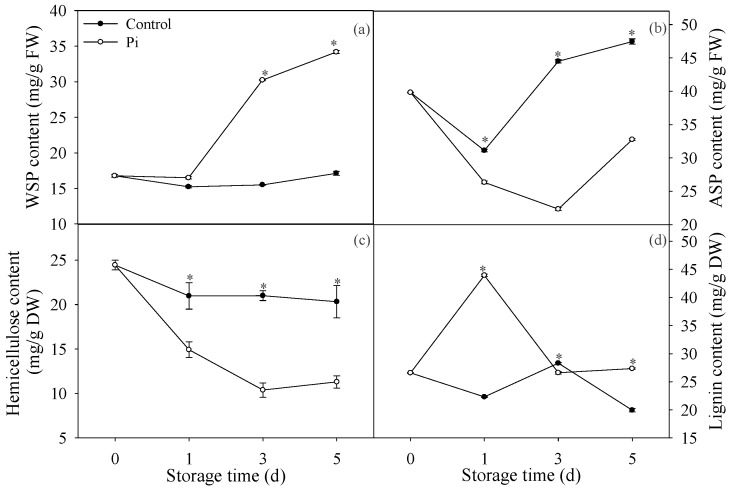
Effects of *P. italicum* infection on water-soluble pectin (WSP) (**a**), acid-soluble pectin (ASP) (**b**), hemicellulose (**c**) and lignin (**d**) of mandarin peel during storage at 25 °C. Each data point represents a mean of three replicate assays ± standard error. Asterisks at the same storage time represented the significant differences (*P* < 0.05). FW: fresh weight; DW: dry weight.

As Figure 1a showed, WSP in control fruit showed no significant changes after five days of storage. In contrast, *P. italicum*-treated fruit showed a sharp increase in WSP content after day 1 (Figure 1a). In addition, the WSP content in *P. italicum*-treated fruit was much higher than that in control fruit during the storage period. In contrast, ASP content in *P. italicum*-treated fruit was much lower than that in control fruit during the storage period (Figure 1b). Hemicellulose content in control fruit decreased slightly at day 1, then did not change during storage (Figure 1c). *P. italicum* inoculation accelerated the decrease of hemicellulose and the hemicellulose contents in *P. italicum*-treated fruit were much lower than those in control fruit during the whole storage period (Figure 1c). For lignin content, *P. italicum* treatment resulted in higher lignin content compared to control, except day 3 (Figure 1d). Altogether, these results showed that *P. italicum* infection could evidently affect pectin, hemicellulose, and lignin contents in the mandarin peel.

### 3.2. Effects of *P. italicum* Infection on H_2_O_2_ Content, ·OH Scavenging Rate, and AsA Content of Mandarin Peel

**Figure 2:**
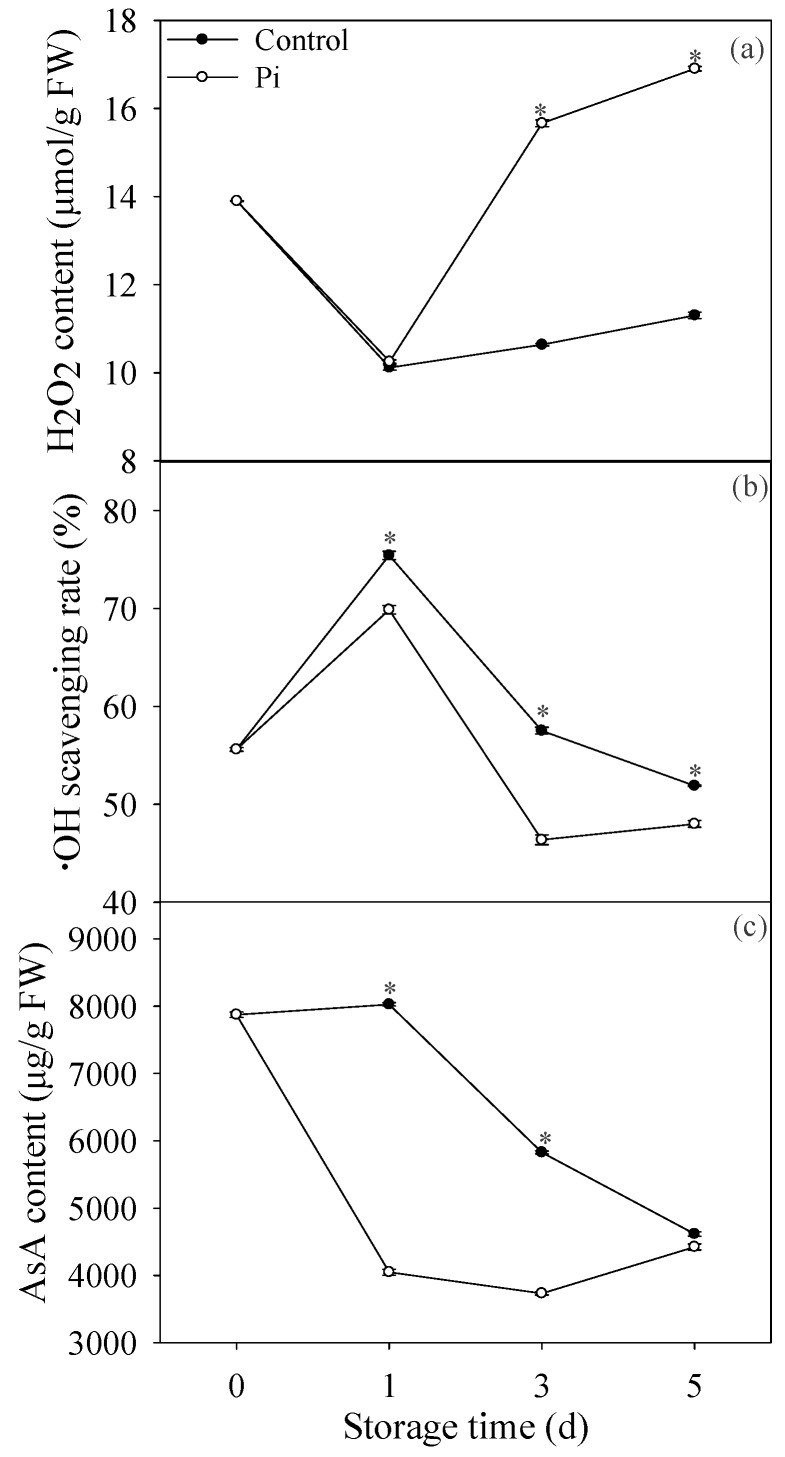
Effects of *P. italicum* infection on H_2_O_2_ content (**a**), ·OH scavenging rate (**b**), and ascorbic acid (AsA) content (**c**) of mandarin peel during storage at 25 °C. Each data point represents a mean of three replicate assays ± standard error. Asterisks at the same storage time represented the significant differences (*P* < 0.05).

As shown in Figure 2a, H_2_O_2_ content decreased first then increased at late storage period. H_2_O_2_ content showed no significance between control fruit and *P. italicum*-infected fruit at day 1. However, from day 3 of storage, *P. italicum* infection significantly increased H_2_O_2_ content compared to control fruit. In contrast, *P. italicum*-infected ‘shatangju’ mandarin fruit had much lower ·OH scavenging rate than control fruit (Figure 2b). For AsA, its content decreased during the whole storage time in control fruit, and *P. italicum* infection accelerated the reduction of AsA content in mandarin peel (Figure 2c).

### 3.3. Effects of *P. italicum* Infection on SOD, CAT, and APX Activities of Mandarin Peel

**Figure 3:**
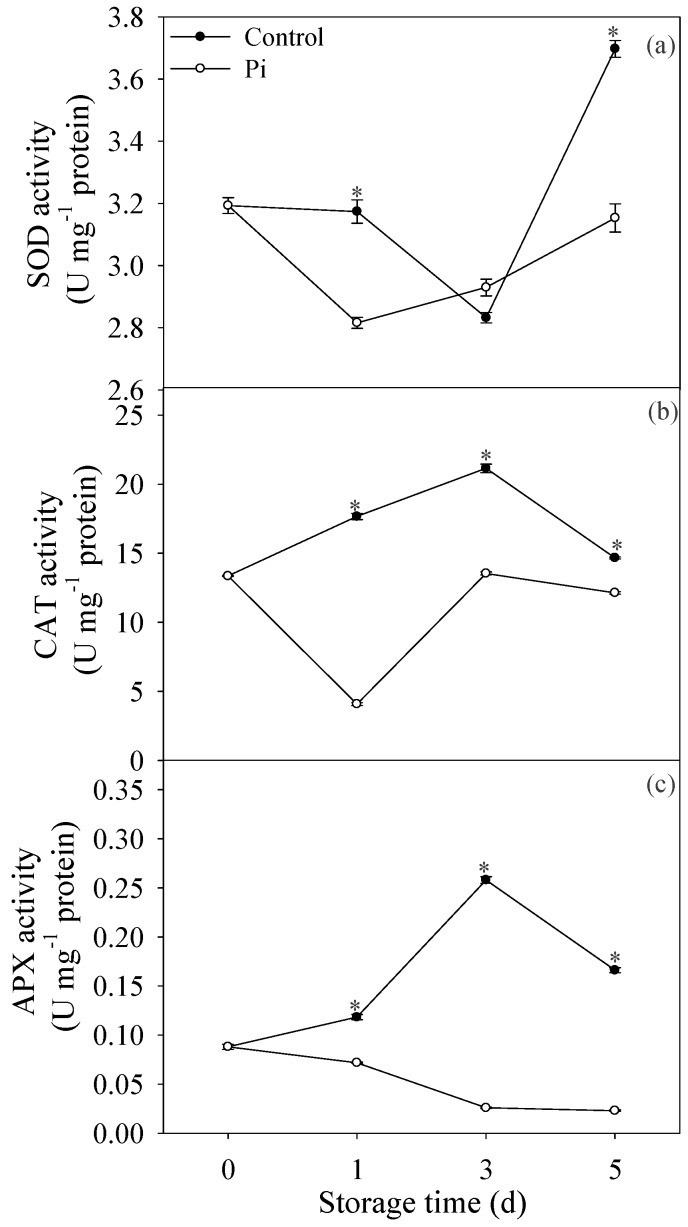
Effects of *P. italicum* infection on enzyme activity of superoxide dismutase (SOD) (**a**), catalase (CAT) (**b**), and ascorbate peroxidase (APX) (**c**) of mandarin peel during storage at 25 °C. Each data point represents a mean of three replicate assays ± standard error. Asterisks at the same storage time represented the significant differences (*P* < 0.05).

Figure 3a showed that SOD activity decreased before day 3 storage, then increased significantly at day 5 in control fruit. Except day 3, the SOD activity in control fruit was higher than that in *P. italicum*-infected fruit (Figure 3a). CAT activity increased at the first three days of storage, then decreased at day 5 in control fruit. In contrast, in *P. italicum*-treated fruit, CAT activity decreased at day 1, then increased during storage. Additionally, Pi treatment resulted in lower CAT activity compared to control fruit (Figure 2b). Similarly, APX activity in *P. italicum*-treated fruit was also much lower than that in control fruit during the storage period (Figure 3c). In all, our results suggested that *P. italicum* infection reduced the antioxidant enzymes activities in mandarin peel.

### 3.4. Effects of *P. italicum* Infection on PG and PME Activities of Mandarin Peel

**Figure 4:**
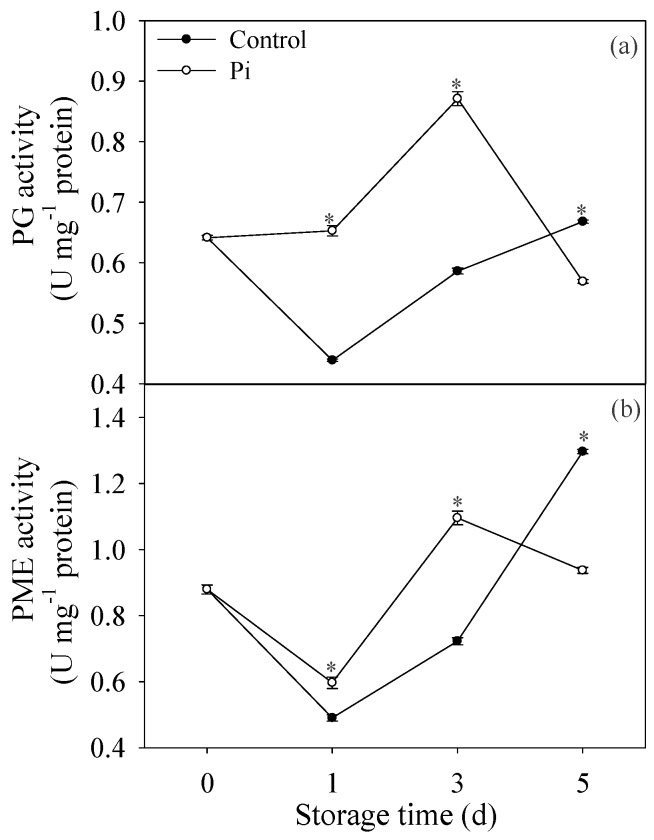
Effects of *P. italicum* infection on enzyme activity of polygalacturonase (PG) (**a**) and pectin methylesterase (PME) (**b**) of mandarin peel during storage at 25 °C. Each data point represents a mean of three replicate assays ± standard error. Asterisks at the same storage time represented the significant differences (*P* < 0.05).

Figure 4a indicated that PG activity decreased at day 1, then increased during storage time in control fruit. Pi treatment enhanced the PG activity compared to control fruit during storage time, except at day 5 (Figure 4a). Similarly, PME activity showed the same pattern as PG in control fruit. Additionally, PME activity in *P. italicum*-treated fruit was much higher than that in the control fruit, except at day 5 (Figure 4b).

The findings indicated that *P. italicum* infection caused a significant increases in activities of cell wall-degrading enzymes including PG and PME in the peel of harvested mandarin fruit, and maintained them at relatively higher levels during storage.

### 3.5. Effects of *P. italicum* Infection on Gene Expression Level of Mandarin Peel

**Figure 5:**
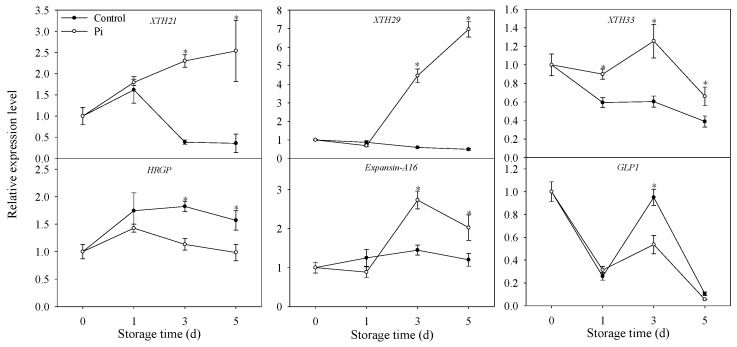
Effects of *P. italicum* infection on the expression levels of cell wall-related genes. Each data point represents a mean of three replicate assays ± standard error. Asterisks at the same storage time represented the significant differences (*P* < 0.05). XTH: xyloglucan endotransglucosylase/hydrolase; HRGP: hydroxyproline-rich glycoprotein; GLP1: Germin-like protein 1.

As shown in Figure 5, the expression of *XTH* genes decreased along with storage time in control fruit. In *P. italicum*-treated mandarin peel, *XTH* genes increased significantly compared to the values at day 0, except *XTH33* at day 5 (Figure 5). Importantly, all the expression levels of XTH in *P. italicum*-treated fruit were much higher than those in control fruit (Figure 5). *HRGP* gene showed same expression pattern in both control and *P. italicum*-treated fruit peel, but with higher expression level in control fruit from day 3. For *GLP1*, though same expression pattern also appeared in control and Pi-treated fruit, the higher expression level was only observed in control fruit at day 3 (Figure 5). The expression level of *expansin-A16* did not change significantly in control fruit during storage, while increased significantly in *P. italicum*-treated fruit (Figure 5). All these results indicated that *P. italicum* inoculation could induce cell wall modification via transcriptional level regulation.

## 4. Discussion

**Figure 6:**
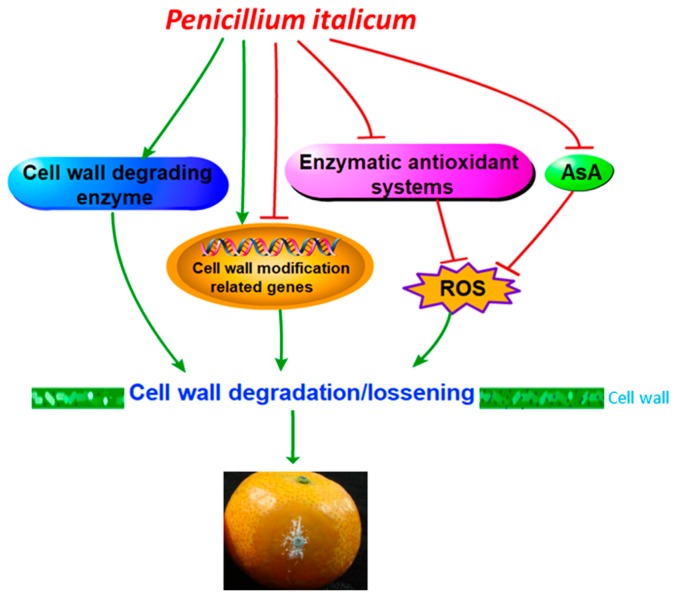
The possible mechanism of *P. italicum* infection inducing cell wall degradation of harvested ‘shatangju’ mandarin fruit. → Represents positive effects while 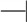 Represents negative effects. AsA: ascorbic acid; ROS: reactive oxygen species.

Fungal disease is considered as the key factor restricting the transportation and marketing of harvested citrus fruit [2]. A major pathogen resulting in the decay of harvested mandarin is *P. italicum*. A better understanding of fruit responses to pathogen attacks is essential to clarify the infection mechanisms of pathogen and develop novel methods to control disease development and improve fruit quality. In the present work, *P. italicum* inoculation on ‘shatangju’ mandarin promoted fruit disease occurrence significantly after storage of 5 days (Figure 6). We then focused on the cell wall changes and its regulatory mechanism of ‘shatangju’ mandarin peel after *P. italicum* inoculation. 

Several studies have demonstrated that cell wall compounds can be affected by fungi infection [29,34]. Pectin is a cell wall polysaccharide in plants, including fruit crops [35] and pectin is an essential compound in the cell wall maintaining the structural integrity [36]. In this study, our results showed that *P. italicum* increased WSP content while decreased ASP content compared to control fruit (Figure 1a,b). These results suggested the enhancement of the dissolving of ASP to WSP after *P. italicum* inoculation, which resulted in solubilization of loosely bound pectin in mandarin peel, which was similar to *C. gloeosporioides* infected valencia orange [29]. Hemicellulose is another polysaccharide component of the primary cell wall and depolymerization of hemicellulose were responsible for fruit softening during ripening [37]. As a consequence of hemicellulosic breakdown, the cellulose–hemicellulose network disruption caused rigidity decrease in fruit cell wall [38]. In this study, we found that hemicellulose content decreased significantly in *P. italicum*-infected mandarin fruit (Figure 1c), indicating the degradation of hemicellulose polysaccharides and cell wall disruption caused by *P. italicum* inoculation. Lignin is another vital cell wall compound preventing the degradation of cell wall polysaccharides [39]. Strangely, in this study, we found that *P. italicum* infection resulted in higher lignin content compared to control, especially at day 1 (Figure 1d). Considering the important role of lignin against pathogens, higher accumulation of lignin might indicate a defense response that retards damage caused by pathogen, which was also reported by Zhao et al. [29]. However, *P. italicum* still infected citrus fruit successfully. We postulated that pectin degradation might contribute more to the *P. italicum* infection than lignin accumulation. Certainly, the complexity of the lignin accumulation in response to *P. italicum* still needs further study.

It has been reported that ROS accumulation is involved in cell wall degradation [17,22]. For example, H_2_O_2_ and superoxide were reported to be involved in the degradation of beta-glucan [17]. In this study, Pi treatment evidently increased the H_2_O_2_ production (Figure 2a), which might contribute to cell wall modification. Furthermore, Xiong et al. [40] also reported that H_2_O_2_ contributed to the regulation of pectin synthesis and PME activity. It is worthy to noting that H_2_O_2_, as a signaling molecule, also plays a central role through the signaling cascade of some transcription factors to regulate cell wall metabolism [41]. Gomez-Ros et al. [41] reported that H_2_O_2_ could induce the genes of the lignin biosynthetic pathway. In this study, the H_2_O_2_ content increased simultaneously with lignin content increase, suggesting lignin synthesis might possibly be activated by H_2_O_2_. However, this needs further investigation in our future research. In contrast, the ·OH scavenging rate was much lower in *P. italicum*-infected fruit than control fruit (Figure 2b), suggesting higher accumulation of ·OH in *P. italicum*-infected fruit. Our previous research indicated that ·OH caused the cell wall disassembly in banana and longan fruit [15,22,28]. The results in the present study confirmed that ROS-mediated cell wall depolymerization and degradation might accelerate the *P. italicum* infection on ‘shatangju’ mandarin fruit. The type of ROS that contributes mainly to the cell wall degradation during fungi infection still needs further clarification.

Reduction of ROS scavenging ability was also reported to contribute to pathogen-induced disease development on fruit [33]. In this study, *P. italicum* infection resulted in lower activities of SOD, CAT, and APX in mandarin peel during the storage period (Figure 3), which was attributed to higher accumulation of ROS, and subsequently contributing to cell wall degradation. In addition, results in the present study also showed that non-enzymatic antioxidant substances, AsA was also lower in *P. italicum*-treated fruit (Figure 2c). Consistent with our results, *L. theobromae* infection also resulted in lower contents of AsA in longan pericarp [33]. In all, reduction of ROS scavenging ability caused by Pi treatment resulted in higher ROS stress in mandarin peel. Furthermore, according to our results, we put forward the possible mechanism of ROS-mediated disease occurrence regarding cell wall degradation (Figure 6). Though we mainly focused on the effect of ROS stress on cell wall degradation in this study, it is important to note that oxidative burst, occurring in plant cells during interactions with pathogens, can stimulate programmed host cell death assisting fungal growth [42,43]. In the present study, the inhibition of the antioxidant system might also result in the activation of the oxidative burst and programmed cell death in ‘shatangju’ mandarin fruit. Further investigation is needed to determine whether ROS mediated programmed cell death was involved in the infection of *P. italicum* on ‘shatangju’ mandarin fruit.

Hydroxyproline-rich glycoprotein was a cell wall related protein playing an important role in cell wall modification and mandarin peel senescence [12]. Higher expression of genes encoding hydroxyproline-rich glycoprotein also contributed to the protection of *Medicago truncatula* against fungal infection [44]. In the present study, the expression level of HRGP was much lower in Pi treatment (Figure 5), indicating cell wall degradation and reduced defense ability in ‘shatangju’ mandarin fruit. Germin-like protein1 (GLP1) is another cell wall-associated protein involved in disease resistance [45]. Lower expression level of GLP1 was also observed in *P. italicum*-infected fruit (Figure 5), suggesting the inhibition of cell wall-mediated disease resistance in mandarin fruit. Another cell wall-modifying protein, expansin could also cause disassembly in the fruit cell wall network [20]. In the present study, *P. italicum* infection increased expansin-A16 expression level compared to control fruit (Figure 5). The increase of *expansin* expression level possibly resulted in the disassembly of the mandarin peel cell wall, which was beneficial for *P. italicum* infection. Altogether, we proposed that non-enzymatic degradation of cell wall enhanced by *P. italicum* infection might be attributed to the reduced resistance of ‘shatangju’ mandarin against *P. italicum*.

The modification in cell wall components is reported to be induced by the action of cell wall hydrolases [46]. For example, PME and PG are two important enzymes that are responsible for breaking pectic polymers. Pectin methylesterase results in the demethylated pectins that can be degraded by polygalacturonase [13]. It was also observed that accumulation of cell wall-degrading enzymes in plant could be induced by pathogen invasion [29]. In this study, PG and PME activities were found higher in infected fruits (Figure 4), resulting in the rotting of citrus fruits. These results are accordance with the observations of Shi et al. [11], who also reported that TS-g-SA could control rotting of grapefruits through the suppression of PG and PME activities. Altogether, the higher activities of PME and PG accelerated the cell wall degradation process in *P. italicum*-treated fruit. It is noteworthy that the *P. italicum*-treated fruit showed sharp declines in the activities of cell wall-degrading-related enzymes (PG and PME) during the late stages of storage (Figure 4). We postulated that this general loss of cell wall related-enzyme activities in the infected fruit reflected cell wall disruption or dysfunction, which were significantly accelerated by fungi infection. Together with the increased activities of PG and PME, higher expression levels of *XTH* genes were also observed in Pi-treated fruit (Figure 5). Considering the important contribution of XTH to cell wall degradation [12], it was postulated that the induction of *XTHs* expression by *P. italicum* might be related to cell wall degradation of the mandarin peel. Therefore, we proposed that the enhanced enzymatic degradation of cell wall might contribute to the successful infection of *P. italicum* on ‘shatangju’ mandarin. In accordance with our results, citrus fruit treated with *Colletotrichum gloeosporioides* also showed enhancement of enzymatic cell wall degradation [29]. In all, we concluded that enzymatic degradation of cell wall was also enhanced by *P. italicum* infection, which might contribute to the successful infection of *P. italicum* on ‘shatangju’ mandarin fruit.

## 5. Conclusions

As shown in Figure 6, the findings in this study indicated that *P. italicum* infected ‘shatangju’ mandarin cultivar through modulating cell wall structure. Cell wall degradation might be attributed to higher accumulation of ROS production caused by inhibited antioxidant activity and lower AsA accumulation in ‘shatangju’ mandarin. Higher expression of *expansin* together with lower expression of *GLP1* also contributed to the non-enzymatic degradation of cell wall of ‘shatangju’ mandarin peel. Additionally, enhancement of enzymatic degradation of ‘shatangju’ mandarin cell wall was observed after *P. italicum* infection. These results provide a new basis for the study of the interaction between *P. italicum* and citrus fruit and potential target to control disease and extend the shelf life of ‘shatangju’ mandarin fruit.

## Supplementary Materials

The following are available online at https://www.mdpi.com/2218-273X/9/4/160/s1, Table S1: Primer sequences of selected genes for qRT-PCR in this study.
