# Supplementary material for: Mechanism of Cell Wall Polysaccharides Modification in Harvested ‘Shatangju’ Mandarin (Citrus reticulate Blanco) Fruit Caused by Penicillium italicum
Source: Biomolecules. 2019 Apr 24;9(4):160. doi: 10.3390/biom9040160 (PMC6523094; doi:10.3390/biom9040160)
Supplement: Supplementary file 1 [file biomolecules-09-00160-s001.pdf]

Supplementary Table S1. Primer sequences of selected genes for qRT-PCR in this study.

Gene name	Forward primer (5'→3')	Reverse primer (5'→3')
XTH21	CAACAACTCTGCTGGCACTGTCACT	CAGTCATCGGCATTCCACAGGCTAG
XTH29	TTCGTCTCCTCCTCAACCGCTTCT	CGTGCCGTGCTCCCATTTCCAT
XTH33	CCAACGGCACAGCACCATTACTACA	CATGAAGCAGGAGCAGAGGCAGAAT
HRGP	GCCTCTTTCCACTACTCCCACCCA	TGGTTACTCCTCCTGCGACTCATCA
Expansin-A16	CCTTCAATCTTCGTCACTGCCACCA	AACCTGATGCCGCCTCTCTTCCT
GLP1	AGCCTGGCAAGACAAGTGGTAACA	AGCCTTTAGGGAACACAAACGAGTCA
Actin	ATCTGCTGGAAGGTGCTGAG	CCAAGCAGCATGAAGATCAA
